# Knowledge and risk perception of pesticide exposure among smallholder farmers of Sembabule district, Uganda

**DOI:** 10.4314/ahs.v26i2.19

**Published:** 2026-06

**Authors:** Evelyn Nakuya, Lesley Rose Ninsiima, Allan Ssembuusi, David Musoke, Victoria Nankabirwa

**Affiliations:** 1 Department of Disease Control and Environmental Health, School of Public Health, College of Health Sciences, Makerere University, Kampala, Uganda; 2 Department of Biosecurity, Ecosystems and Veterinary Public Health, Makerere University, Kampala, Uganda; 3 Department of Epidemiology and Biostatistics, School of Public Health, College of Health Sciences, Makerere University, Kampala, Uganda

**Keywords:** Pesticides, exposure, smallholder farmers, risk perception

## Abstract

**Background:**

Smallholder farmers in Uganda are commonly exposed to high levels of pesticides. Improper handling of these chemicals can cause numerous health risks. Hence, this study assessed the knowledge and risk perception of pesticide exposure among smallholder farmers of Sembabule district, Uganda.

**Methods:**

A cross-sectional study among 278 smallholder farmers was conducted, using a researcher administered questionnaire. Participants were selected using systematic sampling with an interval of each 8th household. A generalized linear model with modified Poisson regression was used to estimate prevalence ratios (PRs) and corresponding 95% confidence intervals (Cls) for factors associated with low-risk perception of pesticide exposure, including sociodemographic and knowledge related-variables were adjusted for in the multivariable model.

**Results:**

Overall, 66.19% (184/278) of the participants had poor knowledge of pesticide exposure, and 54.68% (152/278) had a low-risk perception of pesticide exposure. Low-risk perception of pesticide exposure was associated with lack of concern about negative impacts of pesticide exposure [Adjusted Prevalence Ratio, aPR = 4.96, 95% Cl: 2.13-11.56] and with the perception that pesticides do not affect the environment [aPR = 1.85, 95% Cl:1.22-2.81].

**Conclusion:**

Smallholder farmers had low-risk perception of pesticide exposure. This underscores the need for national farmer-trainings by agricultural extension workers on safe pesticide use, and improvement of existent government pesticide sale regulations.

## Introduction

Pesticides are increasingly used globally to control public health and agricultural pests and diseases, particularly in sub-Saharan Africa (SSA)^1^-^3^. However, pesticide use may turn out to be dangerous to the users through exposure, especially to the most risk populations such as farmers due to lack of knowledge on safe pesticide use, poor pesticide handling behavior for example, not using personal protective equipment (PPE) while handling pesticides^4^. In developing countries, pesticide poisoning has been highly rated as a public health challenge^5^. According to the World Health Organization (WHO), it is estimated that more than 200,000 lives are lost every year in developing countries as a result of pesticide poisoning^6^.

In addition, human exposure to pesticides is associated with several health risks including neurological consequences such as chronic and acute neurological symptoms (including headache)^7^, and mental health issues (including increased depression)^8^. For instance, exposure to glyphosate, a commonly used herbicide in low- and middle-income countries (LMICs) including Uganda, is found to be associated with impaired visual memory^9^, and mancozeb exposure, a fungicide containing manganese, is linked to sleep problems, developmental effects in children, endocrine disruption, Parkinson's disease, and alteration of the immune system response^10^. Coughing, runny nose, dizziness, blurred vision, headache, general body weakness, and itchy skin are some of the common short-term effects of pesticide exposure^11^,^12^.

In Uganda, majority of the farmers use pesticides to increase their crop yields. However, most of the pesticides used are classified as hazardous by WHO^13^. Pesticides account for a case fatality rate of 1.4 (per 100 000) in Uganda, with high percentage cases related to intentional self-poisoning^14^. Occupational exposure to pesticides often occurs through skin contact, inhalation, and ingestion during mixing and spraying of pesticides^15^. Additionally, a study done in Uganda demonstrated that smallholder farmers are occupationally exposed to pesticides as they directly mix, and spray pesticides^2^. Notably, households near farms are also likely to be exposed to pesticides due to drift and vaporization of pesticides during application^16^. Moreover, there is limited regulation, monitoring, and control in sale and use of pesticides in many LMICs, like Uganda which always lead to unprotected exposure among users^17^.

A study done in Uganda on pesticide exposure affirmed that the low level of education, and lack of training on pesticide use among agricultural farmers contributes to their high exposure rates and health risks^18^, aggravated by farmers' inadequate knowledge of the toxicity of pesticides and the health outcomes of pesticide exposure^19^. Therefore, in order to reduce the negative health and environmental pesticide effects, farmers should be sensitized on the importance of adherence to safe pesticide use, handling, spraying techniques, and first aid for pesticide poisoning^20^. To the best of our knowledge, there is limited literature on the risk perception of pesticide exposure among smallholder farmers in Uganda^21^. Previous studies focused on knowledge of smallholder farmers of pesticide use, rather than their risk perception of pesticide exposure^18^, ^22^. Therefore, this study addressed this research gap, and assessed the level of knowledge and risk perception of pesticide exposure among smallholder farmers of Sembabule district. This is an area in Uganda that is dominated by hardly-trained farmers yet cultivate crops with pesticides, rarely use PPE due to socioeconomic constraints, and work in a system with weak pesticide use regulation^23^, ^24^. This study's findings may be widely generalized to similar smallholder farming districts in Uganda.

## Methods

### Study design and area

This cross-sectional study was carried out in 2023 in Mitete parish, Mateete sub-county, Sembabule district, located in the Central region of Uganda. Mitete parish is predominantly a rural agricultural area, entirely depending on rain as the main source of water for agriculture. The parish is subdivided into 17 villages including Kafumu, Kanyogoga A, B and C, Kyabakagga A and B, Nakaseeta, Kalububbu, Lukaka A and B, Kalukungu, Kijju, Nabiyagi A and B and Mitete A, B and C. Mitete is home to 10,196 people which constitutes approximately 4% of the district population, with 2,339 households^25^. Mitete parish was purposively selected for the study because its biggest population is predominantly peasant farmers. Farmers in the area grow crops for both food and cash earnings. Commonly grown crops in the parish include coffee, bananas, millet, and maize^26^. However, passion fruits, cabbage, tomatoes, watermelon and coffee are the most sprayed crops with Highly Hazardous Pesticides (HHPs) active ingredients which increases risk of exposure to farmers^27^. These agrcultural practices, and pesticide use indicators are similar to those in Uganda's central farming communities^28^.

### Study population, sample size, and sampling

This study was conducted among smallholder farmers aged 18 years and above. Smallholder farmers were defined as those with farms less than 2 hectares^18^. The minimum sample of 278 was determined using the Kish and Leslie formula, with a 95% confidence interval, precision of 5% and Z-value of 1.96^29^. Out of the seventeen villages in the parish, ten of them were randomly selected using Microsoft Excel codes. These were corresponding to the first codes, from one to ten, and included Kalububbu, Kalukungu, Kafumu, Kanyogoga B, Kanyogoga C, Kijju, Kyabakagga A, Kyabakagga B, Mitete B and Nakaseeta.

In each of these ten villages, lists of smallholder farmers were generated with assistance from the local leaders. Systematic random sampling was done due its efficiency, feasibility and ability to give a probability-based sample since the existing household lists were complete, but with few data collection resources. Using systematic random sampling, 28 households per village were expected on average, from a total of 2,339 households in all villages, to achieve a sample size of 278. The sampling interval (k) in each village was calculated as, k = total listed households in the village divided by the average number of households per village (n = typically 28, but was slightly adjusted in a few villages to achieve proportionality). This provided a sampling interval of approximately each 8th household in majority of the villages (for example a village that had 224 listed households: k = 224/28 = 8). In each village, the first participant was randomly selected, and the rest of the participants were selected using systematic random sampling, with a random starting point (random number between 1 and k), after which every kth household on the village list was selected until the required sample size for that particular village was obtained. From each household, one eligible participant was chosen to take part in the study. In cases where one household had more than one eligible participant, random sampling was done to choose one study participant.

### Data collection tools and procedures

This study employed a structured questionnaire which was designed in English and translated into Luganda, which is the most spoken ethnic language in the study area. The questionnaire explored participants' knowledge and risk perceptions of pesticide exposure, as well as their socio-demographic characteristics. The questionnaire was uploaded on Kobo collect application software that was used for data collection. This was delivered through a researcher administered questionnaire and took approximately 30 minutes to complete. Pre-testing of the questionnaire was done in a similar setting among smallholder farmers of Kayunga parish, Mateete sub-county, Sembabule district to ensure its easiness and comprehensiveness. After pre-testing, questionnaire reliability was done using the pilot test feedback, to identify any errors or inconsistences which were corrected before actual data collection. Completed questionnaires were cross-checked every single day of data collection to ensure that errors were rectified before leaving the field. Additionally, social desirability bias was limited by ensuring that all participants answered the questionnaires independently with limited external influence.

### Variables and measurements

#### Outcome variable

The outcome was risk perception of pesticide exposure. Risk perception was defined as how the participants perceived the likelihood of harm and severity of consequences when exposed to pesticides (through inhalation, oral, skin and eye contact). This was measured as a binary outcome (high-risk perception and low-risk perception), assessed using six questions on; the thought of wearing personal protective equipment while spraying, concern about negative impacts of pesticide exposure, ability of pesticides to affect the environment, the importance of observing weather conditions before spraying and appropriateness of entering recently sprayed fields. The above questions had responses, “Yes” = 1, “No”, and “Don't know” = 0. Another question was on the least conducive weather for spraying which had options (Windy = 1, Rainy = 2, Sunny = 3, Not sure = 4). To obtain the average risk perception for the least conducive weather condition for spraying, participants who responded: Windy and Rainy were coded “1”, whereas Sunny and Not sure: were coded “0”. The total scores for risk perception were calculated by summating the raw scores of the 6 questions ranging from 0 to 6, with an overall higher score indicating a high-risk perception. A cut-off level of more than or equal to 3 for correctly answered questions, was set for a high-risk perception, otherwise the rest had low-risk perception.

#### Independent variables

Knowledge was assessed using nine questions related to awareness of safe pesticide use or handling, source of pesticide information, understanding of pesticide label information, training on pesticide use, awareness of pesticide exposure routes, integrated pest management, toxicity color codes on pesticide packages, known pesticide exposure routes, and symptoms. The knowledge scores for each participant were used to compute a total score of 9. The total score for knowledge was obtained by summating the raw score of each item and ranged from 0 to 9, with an overall greater score indicating good knowledge. A cut-off level of more than or equal to 5 of correctly answered questions, was set for categorizing good knowledge, otherwise the rest had poor knowledge. Socio-demographic characteristics assessed included sex, age in years, highest academic qualification, marital status, occupation, farming type practiced and farming experience in years.

### Data management and analysis

Data was collected using Android-enabled mobile phones loaded with Kobo collect application software. Collected data was synchronized onto the server every day and only the Principal Investigator had access to it. The data was cleaned in Microsoft Excel 2019 and analyzed using STATA 14.0 statistical software. All missing and non-applicable responses were excluded from the analysis. Descriptive analyses such as frequencies and their respective percentages were performed for categorical data, and the mean and the respective standard deviation (SD) were done for numerical data. To assess the outcome variable (risk perception of pesticide exposure), and each explanatory variable, generalized linear model of modified Poisson regression was done, providing Prevalence Ratios (PRs) and corresponding 95% confidence intervals (Cls). Variables with p<0.05, and other socio-demographic characteristics were all added to the multivariable analysis model to ascertain the significant variables for each outcome. This was conducted using a stepwise backward elimination method until only significant predictors were retained in the model. The statistical significance levels were two-sided at p<0.05.

## Ethical considerations

Approval to conduct the study was obtained from Makerere University School of Public Health as part of the Bachelor of Environmental Health Science programme. Administrative clearance was obtained from the local leaders of selected villages of Mitete parish, Sembabule district. The study objectives were explained to participants, written informed consent was obtained before data collection, and their names were not recorded.

## Results

### Socio-demographic characteristics

A total of 278 participants took part in the study. These were predominantly males 58.27% (162/278), aged between 31-40 years 36.69% (103/278), and with a mean age of 36 years (SD 11.90). Half of the participants 50.00% (139/278) had attained a primary level of education, the majority 75.54% (210/278) were married/cohabiting, and farmers by profession 87.77% (244/278). Almost all the participants 90.65% (252/278) practiced subsistence farming, and 91.37% (254/278) had spent more than 10 years as farmers (Table 1).

**Table 1:** Socio-demographic characteristics of the participants

Variable	Frequency (n=278)	Percentage (%)
**Sex**		
Male	162	58.27
Female	116	41.73
**Age (years)**	Mean (SD) = 36.40 (11.90)	
18-30	46	16.55
31-40	103	36.69
41-50	64	23.38
51-60	36	12.95
>60	29	10.43
**Highest Academic Qualification**		
Never attended	92	33.09
Primary	139	50.00
Secondary	36	12.95
Tertiary	11	3.96
**Marital status**		
Married/Cohabiting	210	75.54
Divorced/Separated	33	11.87
Widowed	25	8.99
Single	10	3.60
**Occupation**		
Salary employee	4	1.44
Self employed	6	2.16
Farmer	244	87.77
Casual laborer	20	7.19
**Farming type practiced**		
Subsistence	252	90.65
Commercial	26	9.35
**Farming experience (years)**		
1-3	7	2.52
5-10	17	6.12
>10	254	91.37

### Knowledge of pesticide exposure

Among the participants, 55.76% (155/278) were not aware of safe pesticide use or handling, and 86.33% (240/278) did not have any training on pesticide use. Over half of the participants 51.44% (143/278) were aware of pesticide exposure routes while 60.43% (168/278) were not aware of biological or other forms of integrated pest management. Additionally, 52.88% (147/278) knew potential pesticide exposure symptoms, 60.07% (167/278) did not understand pesticide label information, and 73.74% (205/278) were not aware of toxicity color codes on pesticide packages. Overall, 66.19% (184/278) had poor knowledge of pesticide exposure (Table 2).

**Table 2:** Knowledge of pesticide exposure

Variable	Frequency (n=278)	Percentage (%)
**Awareness of safe pesticide use or handling**		
Yes	123	44.24
No	155	55.76
**Source of pesticide information**		
Agricultural extension officers	8	2.88
Agrochemical retailers	94	33.81
Other farmers	51	18.35
Previous experience	58	20.86
Pesticide containers	52	18.71
None of the above	15	5.40
**Had training on pesticide use**		
Yes	38	13.67
No	240	86.33
**Awareness of pesticide exposure routes**		
Yes	143	51.44
No	135	48.56
Knows possible pesticide exposure routes*		
Inhalation	79	56.03
Skin/Dermal contact	95	67.38
Oral	47	33.33
Eye contact	49	34.75
**Awareness of biological or other forms of integrated pest management**		
Yes	110	39.57
No	168	60.43
Knew potential pesticide exposure symptoms**		
Yes	147	52.88
No	131	47.12
**Understood pesticide label information**		
Yes	111	39.93
No	167	60.07
**Awareness of toxicity color codes on pesticide packages**		
Yes	73	26.26
No	205	73.74
**Overall knowledge**		
Poor	184	66.19
Good	94	33.81

* Multiple response questions

** Symptoms included skin irritation, salivation, nausea, blurred vision and vomiting

### Risk perception of pesticide exposure

More than half of the participants 58.63% (163/278) thought of wearing PPE while spraying, and 54.68% (152/278) had concerns about the negative impacts of pesticide exposure. The majority of participants 89.93% (250/278) positively perceived the importance of observing weather conditions before spraying. Overall, 54.68% (152/278) had a low-risk perception of pesticide exposure (Table 3).

**Table 3:** Risk perception of pesticide exposure

Variable	Frequency (n=278)	Percentage (%)
**Thought of wearing PPE while spraying**		
Yes	163	58.63
No	115	41.37
**Had concerns about the negative impacts of pesticide exposure**		
Yes	152	54.68
No	126	45.32
**The ability of pesticides to affect the environment**		
Yes	124	44.60
No	135	48.56
Did not know	19	6.83
**Importance of observing weather conditions before spraying**		
Yes	250	89.93
No	20	7.19
Did not know	8	2.88
**The least conducive weather for spraying**		
Windy	12	4.32
Rainy	224	80.58
Sunny	21	7.55
Not sure	21	7.55
**Appropriateness of entering recently sprayed fields**		
Yes	153	55.04
No	125	44.96
**Overall risk perception**		
Low	152	54.68
High	126	45.32

### Factors associated with low-risk perception of pesticide exposure

Using modified Poisson regression analysis, participants who were not concerned about the negative impacts of pesticide exposure were 4.96 times more likely to have a low-risk perception of pesticide exposure compared to those concerned [Adjusted Prevalence Ratio, [aPR] = 4.96, 95% Cl: 2.13-11.56, p-value = <0.001]. Additionally, participants who reported that pesticides were unable to affect the environment were 1.85 times more likely to have a low-risk perception of pesticide exposure compared to those who knew [aPR = 1.85, 95% Cl:1.22-2.81, p-value = 0.004]. Participants who reported that there was no importance of observing the weather conditions before spraying were 1.52 times more likely to have a low-risk perception of pesticide exposure compared to those who agreed [aPR = 1.52, 95% Cl:1.17-1.97, p-value = 0.002] (Table 4).

**Table 4:** Factors associated with low-risk perception of pesticide exposure

Variable	Risk perception of pesticide exposure		Crude PR 95% Cl	P-values	Adjusted PR 95% Cl	P-values

	Low (n = 152)	High (n = 126)				
**Sex**						
Male	82(50.62)	80(49.38)	1		1	
Female	70(60.34)	46(39.66)	0.80(0.61-1.06)	0.116	1.12(0.91-1.37)	0.284
**Age (years)** 20-30	36(78.26)	10(21.74)	1		1	
31-40	47(46.08)	55(53.92)	2.48(1.39-4.42)	**0.002**	0.99(0.65-1.51)	0.969
41-50	33(50.77)	32(49.23)	2.26(1.24-4.14)	0.008	0.87(0.57-1.35)	0.542
51-60	19(52.78)	17(47.22)	2.17(1.13-4.16)	0.019	0.89(0.56-1.42)	0.637
>60	17(58.62)	12(41.38)	1.90(0.95-3.83)	0.720	0.99(0.59-1.67)	0.975
**Awareness of safe pesticide use or handling**						
Yes	102(82.93)	21(17.07)	1		1	
No	50(32.26)	105(67.74)	3.97(2.65-5.95)	**<0.001**	1.08(0.78-1.50)	0.644
**Had training on pesticide use**						
Yes	31(81.58)	7(18.42)	1		1	
No	121(50.42)	119(49.58)	2.69(1.36-5.33)	**0.004**	1.16(0.77-1.74)	0.468
**Aware of pesticide exposure routes**						
Yes	122(85.3)	21(14.69)	1		1	
No	30(22.22)	105(77.78)	5.30(3.53-7.95)	**<0.001**	1.30(0.93-1.81)	0.128
Knew possible pesticide exposure symptoms**						
Yes	130(88.44)	17(11.56)	1		1	
No	22(16.79)	109(83.21)	7.19(4.57-11.33)	**<0.001**	0.63(0.29-1.38)	0.252
**Understood pesticide label information**						
Yes	90(81.08)	21(18.92)	1		1	
No	62(37.13)	105(62.87)	3.32(2.22-4.97)	**<0.001**	0.90(0.68-1.19)	0.467
**Aware of toxicity color codes on pesticide packages**						
Yes	66(90.41)	7(9.59)	1		1	
No	86(41.95)	119(58.05)	6.05(2.96-12.38)	**<0.001**	1.97(0.99-3.93)	0.053
**Had concerns about the negative impacts of pesticide exposure**						
Yes	136(89.47)	16(10.53)	1		1	
No	16(12.70)	110(87.30)	8.29(5.19-13.26)	**<0.001**	4.96(2.13-11.56)	**<0.001**
**The ability of pesticides to affect the environment**						
Yes	106(85.48)	18(14.52)	1		1	
No	42(31.11)	93(68.89)	4.75(3.05-7.39)	**<0.001**	1.85(1.22-2.81)	**0.004**
Did not know	4(21.05)	15(78.95)	5.44(3.34-8.85)	**<0.001**	1.89(0.93-3.84)	0.080
**Importance of observing weather conditions before spraying**						
Yes	150(60.00)	100(40.00)	1		1	
No	2(10.00)	18(90.00)	2.25(1.82-2.78)	**<0.001**	1.52(1.17-1.97)	**0.002**
Did not know	0(0.00)	8(100.00)	2.5(2.15-2.91)	**<0.001**	1.43(0.69-2.96)	0.337
**Thought of wearing PPE while spraying**						
Yes	140(85.89)	23(14.11)	1		1	
No	12(10.43)	103(89.57)	6.35(4.32-9.32)	**<0.001**	1.66(1.05-2.62)	0.030

**Key; PR:** Prevalence Ratio, **Cl:** Confidence Interval

** Symptoms included skin irritation, salivation, nausea, blurred vision and vomiting

## Discussion

This study assessed the level of knowledge and risk perception of pesticide exposure among smallholder farmers of Sembabule district. Our study revealed a poor overall knowledge of pesticide exposure among smallholder farmers. This could be attributed to their low level of education and limited training on safe pesticide use and handling such as by the district agricultural extension workers. A recent related study in the Lake Albert Crescent, southwestern, and eastern highland districts of Uganda found a low level of knowledge among potato farmers, evidenced by their inability to explain the toxicity labels on the pesticide containers as well as the inadequate training on pesticide use by agricultural extension workers^22^. This calls for the enhancement of farmers' knowledge on possible measures to mitigate pesticide exposure through financing of training by government agricultural extension workers and Non-Governmental Organizations (NGOs).

Our findings show that the overall risk perception of pesticide exposure was low. This could be attributed to limited awareness of the associated short-term and long-term effects of pesticide exposure among smallholder farmers. Similarly, a study done among farmers in Wakiso district in central Uganda found that they would not care about the health risks associated with exposure to pesticides to themselves and the end users of their products^30^. This finding highlights the need to strengthen the already existing policies that regulate pesticide use and enforce pesticide control measures in Uganda.

The study suggests an association between poor knowledge and low-risk perception of pesticide exposure among smallholder farmers, which eventually results into unsafe practices. This is similar to a recent study done in Uganda which found that increased awareness through an educational intervention resulted into a high-risk perception of pesticide exposure among smallholder farmers^31^. This may be attributed to the theory that increased knowledge on pesticide exposure increases perceived severity. These insights inform national agricultural extension programs aimed at increasing safe pesticide use and application techniques by smallholder farmers to reduce pesticide exposure, which is associated with human health and environmental risks.

The study findings demonstrate that participants who were not concerned about the negative impacts of pesticide exposure were more likely to have a low-risk perception of pesticide exposure. This is consistent with studies done in SSA which found that farmers had a low-risk perception towards pesticide-stained crops^32^,^33^. This calls for promotion of local pesticide control initiatives including use of integrated pest management and non-synthetic pest control measures among smallholder farmers to reduce the consequences of chronic exposure to pesticides such as cancer, skin disease, neuropathy, and blindness.

In addition, participants who reported that it was not important to observe weather conditions before spraying were more likely to have a low-risk perception of pesticide exposure. This is in agreement with findings from an earlier study done in Ethiopia which showed that farmers who did not observe weather conditions before spraying and sprayed against the wind were significantly exposed to pesticides^4^. Ideally, farmers are supposed to observe the weather conditions before spraying and ensure that they spray against the wind direction to reduce exposure to pesticides while spraying^6^. This finding emphasizes the need to increase interventions such as awareness campaigns among smallholder famers, on the importance of observing weather conditions before spraying and the risk of the active ingredients in pesticides on their health and surrounding households.

In this study, reporting that pesticides were unable to affect the environment was associated with a low-risk perception of pesticide exposure. This result was also evident in a recent study conducted among rural farmers in Nigeria which established an association between pesticide exposure with environmental effects^34^. This implies that farmers lack technical knowledge on the proper usage of pesticides. As a result, they are left to make their own decisions on handling of pesticides and deal with pesticide exposure outcomes. This finding highlights the need for technical support by agricultural extension workers and NGOs on the safe use of pesticides, protection from pesticide exposure, and associated risks to human health and the environment among smallholder farmers.

This study did not find an exposure-dependent effect with low-risk perception of pesticide exposure and thought of wearing PPE while spraying. However, a related study done in Uganda found a relationship between PPE use and low-risk perception of pesticide exposure^35^. This may be associated with the small sample size used in this cross-sectional study, and future research should be done including broader randomized controlled trials to examine this causal pathway. PPE including overalls, gloves, eyeglasses, gumboots, nose and face masks have been documented to reduce pesticide exposure, protecting farmers from pesticide health effects, thus saving medical expenses^36^. However, socioeconomic constrains including the high cost of PPE lead to sub-optimal use among smallholder farmers, consequently a potential barrier to improved pesticide safety.

## Conclusion

This study provides information on risk perception of pesticide exposure among smallholder farmers. This baseline information can further be utilized by randomized controlled trials and prospective observational studies to understand the causal pathway between these potential predictors and risk perception of pesticide exposure. The study findings also highlight the need for national farmer-training programs by agricultural extension workers, agrochemical retailers and NGOs on safe pesticide handling measures, and improvement of existent government regulations on pesticide sale and application standards in Uganda. The results from this study could inform policymakers in developing interventions needed to reduce pesticide exposure among smallholder farmers including promotion of integrated pest management and non-synthetic pest control measures.

## Strengths and Limitations

The strength of this study is that it is one of the few studies assessing knowledge and risk perception of pesticide exposure among smallholder farmers, and provides basic knowledge on proper use of pesticides, and risks associated with pesticide exposure in similar settings. In addition, we believe that the robust data analysis explores the relationship between the low-risk perception of pesticide exposure and other explanatory variables. Some limitations worth noting include knowledge and risk perception of pesticide exposure were assessed using a questionnaire which may lead to recall bias. Also, responses about PPE use were self-reported, resulting into a potential of social desirability bias. However, this was limited by ensuring that all participants answered the questionnaires independently with limited external influence. Secondly, being a cross-sectional study, the information gathered is a snapshot of what is happening only at that point of time.

## Data Availability

All relevant data is included in this paper.

## List of abbreviations

aPR: Adjusted Prevalence Ratio
Cls: confidence intervals
HPPs: Highly Hazardous Pesticides
LMICs: low- and middle-income countries
Mt: million tons
NGOs: Non-Governmental Organizations
PRs: Prevalence Ratios
SD: standard deviation
SSA: sub-Saharan Africa
WHO: World Health Organization
